# Gynecological and Obstetrical Society of Baltimore

**Published:** 1891-06

**Authors:** William S. Gardner

**Affiliations:** Secretary; 712 N. Howard St.


### Society Reports.

### GYNECOLOGICAL AND OBSTETRICAL SOCIETY OF BALTIMORE.

March meeting.

DR. HENRY W. WILSON, President, in the chair.

DR. HOWARD A. KELLY read a paper on the technique of the Cæsarean section, described in a series of steps, from the selection of the case down to the after treatment. The relative and absolute indications were described. The Porro operation was rejected, excepting under special peculiar circumstances ; for example, when there was good reason to suspect septic infection, as after prolonged efforts at delivery, at turning, or the use of the forceps ; also in cases of large tumors occupying the body of the uterus, or in some cases of cancer, or in uncontrollable hemorrhage from the placental site. Thus limited, the conservative operation and the Porro operation are mutually exclusive, not occupying the same field.

It is a serious surgical error to mutilate a woman by performing the Porro operation where special indications do not exist.

The mortality of the Porro operation is fully as great and probably greater than that of the conservative.

In a healthy case, free from sepsis, with unruptured membranes, it is not necessary to deliver the uterus from the abdomen before incising it and delivering the child. It is rarely necessary to use any constricting ligature around the cervical end of the uterus. Excessive hemorrhage from the placental site or the margin of the wound can very well be temporarily controlled by constricting the cervix with the hands of an assistant.

The uterine suture consists of deep sutures, embracing the peritoneum and muscularis, but not the decidua. About ten such sutures are needed. Between each of these deep sutures, half deep sutures can be passed, securing perfect coaptation of the peritoneal surfaces. The sero-serous sutures are not necessary in cases free from any suspicion of infection. In such clean cases, the uterus is dropped back into the abdomen and covered with the omentum. If there exists a slight suspicion, it is of advantage to draw the omentum down behind the uterus, thus favoring the discharge of any septic material through the lower angle of the wound.

Drainage of the pelvic cavity cannot be efficiently carried out. The abdominal wound must be concealed by a dressing of snowy cotton dissolved in alcohol and ether, containing one part bichloride to 16,000. A little strip of gauze is laid over the wound saturated with this solution. This adheres until it is time to take the sutures out, concealing the wound and preventing contamination from the outside much better than many layers of gauze and cotton. The baby should be allowed to nurse as soon as the mother has thoroughly recovered from the anæsthetic.

The vagina should not be douched out as a matter of routine. The vaginal outlet should be secured from the introduction of sepsis from without by separating the labia and throwing into the vulvar orifice a drachm of powdered iodoform and boric acid (2 to 7). A cotton pad loosely applied to the vulva should be changed as often as soiled by the discharges. The patient thus passes through a perfectly normal puerperium.

DR. CHAS. P. NOBLE: In the technique of the operation laid down by Dr. Kelly, reference has been made to typical cases. In such cases I agree entirely with what he has said. But all cases are not typical. I will report an unique case upon which I did the Cæsarean section recently.

Dr. Kelly had operated in a previous pregnancy. As a result of the previous operation there remained a fistula opening from the uterine cavity through the abdominal wall. Notwithstanding this fistula, she became pregnant, and for several weeks the amniotic bag protruded into the opening, so that there was nothing between the fœtus and the outer world but the thin amniotic sac.

This sac ruptured at the thirty-third week. The woman had a generally contracted pelvis, besides having a large mass of scar tissue behind the cervix, left from her previous Cæsarean labor. Had spontaneous labor been possible, the fœtus would have escaped through the fistula and not *per vaginam*. In view of the conditions, I thought Cæsarean section preferable to delivering the mutilated fœtus *per via naturales*.

The finger was inserted into the uterus through the fistula, and, with this as a guide, the incision was made through the region of utero-abdominal.

Sufficient room not being afforded for delivery, the peritoneal cavity was opened and the uterine incision lengthened. The living fœtus was then delivered.

The placenta and membranes were firmly adherent, and were closely peeled off. To control bleeding during this time it was necessary to insert the uterus through the abdominal incision, to enable the assistant to grasp the lower segment.

The patient passed through a perfectly normal puerperium, and is now quite well and soundly healed.

This case is entirely unique in its condition and in the technique of the operation.

Three cases of Cæsarean section have been observed by me, all having made good recoveries. When the operation is done at the proper time, and after the method described by Dr. Kelly, I am sure this result will be quite uniform.

The essentials of success are:

1. Operation at the proper time, before labor, or at the beginning of labor.

2. Rapidity in operating.

3. Accurate suturing.

4. Asepsis.

With reference to suturing, I believe that the Lembert suture, as ordinarily described, is purely theoretical. The peritoneum will not hold a suture. Operators have unconsciously included the deeper tissues in the so-called Lembert suture. An important point, not generally recognized in country, is, that the diagnosis should be made in the last weeks of pregnancy, and under ordinary circumstances the operation be decided upon and done at the close of pregnancy before labor sets in, or immediately thereafter. I would not do the modern Cæsarean section in a case which had been tampered with by efforts to deliver with the forceps or by version ; but in such cases would prefer the operation. In Philadelphia in the last four years, twelve Cæsarean sections have been done, and ten mothers have recovered. One that died had pneumonia at the time of the operation. The other case was one in which the surgeon at the same time removed a fibroid tumor.

DR. B. B. BROWNE: I think all the procedures recommended are in the main correct, and are in accordance with the rules and suggestions laid down five or six years ago by Garrigues, Sænger and Leopold. These should be carried out in ideal cases, but unfortunately we meet with many complications which must be dealt with as they occur.

Having recently performed the operation myself and looked up the literature and technique of the subject, I was surprised to find that we can to-day make but little improvement or change for the better.

In 1886 Sænger had operated four times, saving all the women and children. Dr. Leopold had operated nine times and lost one woman, saving all the children.

DR. T. A. ASHBY: I wish to congratulate Dr. Kelly on his brilliant success with the Cæsarean section. This success is convincing proof of what can be done when the section is instituted under proper conditions and at a proper time.

The future of the operation rests upon a proper and judicious selection of the case, and upon an immediate resort to the section before other methods of delivery have been attempted and abandoned.

I doubt whether the Cæsarean section, under such conditions, will give a higher mortality than the ovariotomy of ten or fifteen years ago.

The technique of the section is simple enough, and certainly its mechanical execution is not as difficult as that necessitated in the removal of many conditions of tubal and ovarian disease.

Hemorrhage is not large, and it is easily controlled. Septic processes should not follow if strict aseptic precautions are observed.

The progress of the section as a substitute for other methods of delivery, rests upon an early and clear recognition of the pelvic measurements and a prompt acceptance of this method as the proper procedure in the given case. When this is done the success of the section is not compromised by unfortunate interferences in other directions. When we have obtained the statistics of this class of cases, we are in a position to compare the mortality of the section with other operative methods.

DR. W. P. CHUNN: I did not hear the first part of the history of the case, but think I would have removed the ovaries or tied the Fallopian tubes to prevent future conception. It is hard to say just what operation should be done.

DR. NOBLE: In doing a Cæsarean section I would not touch the ovaries and tubes as Dr. Chunn spoke of doing, but would do nothing to prolong the operation. Tying of the tubes would probably cause salpingitis. This objection is purely theoretical. So far as I know this has been done only twice—once in England and once in America.

DR. BRINTON: I have been for some years interested in measuring the pelvis of women. Very often we go to labor cases without knowing anything about the condition of the pelvis.

With the hospital surgeon, who has the best facilities, the Cæsarean operation will undoubtedly be the best in cases of extreme pelvic contraction. But with the average practitioner what is best? I think that, with these physicians, craniotomy will hold the place.

In speaking of craniotomy "holding its place," I referred to those cases of pelvic contraction when the child could be extracted without harm to the mother, say from 1¾ to 3 inches.

DR. T. A. ASHBY: I must offer an apology for presenting a series of experiences which are familiar to all who have done much intra-abdominal work.

I have brought these charred remnants of tubal and ovarian inflammation before the Society to invite discussion, not to exhibit anything original. They represent nearly every phase of intra-pelvic inflammation, and illustrate the various degenerative conditions which are found in the pelvis after an inflammatory fire has passed over these tissues. Of the nine specimens here presented, removed from the same number of cases, no two are alike.

In one case the tube has received the brunt of the attack; in another the ovary is involved in abscess cavities, whilst in the third both tube and ovary are tied up in a knot of adhesive inflammation, and so on through the series.

The clinical histories of these cases would be exceedingly interesting did time admit of a recital, but I shall not tax your patience with details.

We have the same old story in all of these cases save two—one the large specimen of a tubal sac of uncertain origin, probably an interrupted tubal pregnancy of long standing, and the other the remnants of a catarrhal salpingitis and ovaritis with intra-pelvic adhesions. Of the other seven specimens the origin of the condition is of chief interest in this connection, since they explain to my mind the essential factor in the production of the specimen here presented. Each of these women have borne one or more children; in each case the history of the intra-pelvic trouble dates from the last lying-in period which was accompanied with mild or severe symptoms of child-bed fever. In each of these women there was an old lacerated cervix, in some more pronounced than in others. The histories of these cases, as far as they can be made out, and can be interpreted, tell the simple story. During labor a cervical tear occurred; in this wound septic material gained a lodgement, a septic process was established which extended from the cervix to the cavity, from the cavity to the tubes and from the tubes to the intra-pelvic peritoneum.

The severity of the symptoms in each case must have borne some relation to the septic process and to the tissues involved, though no way is offered for verifying this statement. We simply find the results in general destruction of the tube or ovary, or of both, and the inference is that drainage was severed and pus escaped, leaving no remnants of this character behind except in two of the specimens, in which I found pus cavities in the ovary, containing each a drachm or more of pus.

These cases illustrate the fearful havoc which a septic process following parturition may occasion among the pelvic organs. A little fire kindleth a mighty conflagration is literally true in more respects than one. In an experience with other cases I have observed this septic process in its very beginning when limited to the cervix and cavity, and I have seen the lying-in woman's temperature fall from 103 degrees to normal within twelve hours after thorough cleaning and disinfection of the cervix and cavity in these cases, and a complete arrest of the process before the tubes were involved. In another case I have seen tubal and general pelvic peritonitis in active force following immediately the injections in the cervix and cavity. This experience convinces me, despite all other theoretical teachings, that we have in the lying-in state an explanation of those intra-pelvic diseases which render the lives of so many women useless and oftentimes utterly miserable. How is it necessary that the lying-in period should be surrounded with extra hazard, high temperature and severe pain. Aseptic endometritis following parturition may run a very mild and low grade course, and still result in sub-involution, salpingitis, pelvic adhesions, and other intra-pelvic conditions which impair the normal functions of these organs.

The lesson clearly taught by such experience is that aseptic conditions should be enforced in every case of labor; that the least suspicion of sepsis should lead to immediate investigation of the uterine cervix and cavity with a view to thorough cleaning and arrest of the septic process. If this be done—as I have done it in a number of cases, seen with medical friends in consultation—we can cut short a sepsis and arrest a condition which will surely extend to the tubes and pelvic peritoneum in the absence of prompt attention.

DR. B. B. BROWNE: The fact that laceration of the cervix is so frequently found in married women suffering from tubal disease is, I think, because the purulent discharge from the uterus passing over the torn surfaces prevents their union, while the septic material also extends to the tubes. When there is no septic material in the uterus the lacerated surfaces readily unite, and the tubes are not affected.

DR. J. WHITRIDGE WILLIAMS: The specimens exhibited represent a class of cases that are very common, and which will become more so as we become more expert in bimanual examination. Indeed, to a skillful palpator it almost seems that the majority of women examined have more or less tubal or ovarian disease. The specimens are particularly interesting to me because I have studied carefully the pathology of a large number of similar cases.

The etiology in many cases is doubtful, but most observers appear to cling to Noegerrath's theory of latent gonorrhœa. Examination of the pus in cases of pyosalpinx brings forward most interesting facts. For in most cases it is impossible to discover any species of bacteria, either under the microscope or by culture methods, which shows that the bacteria which caused the trouble have long since died, for closed pus cavities are not particularly favorable for the growth of organisms. In two cases we found undoubted gonococci, and in a case following an imperfect abortion the streptococcus, and in another case the staphylococcus aureus.

Clinically, the cases due to the pus organisms are much more acute and virulent than those due to the gonococcus. These results correspond with those of Zweifel, of Leipzig, who has just published his observations. He also found the gono- and streptococcus, but not the staphylococcus. In one of his streptococcus cases the subject was an undoubted virgin, and he accounted for the infection by an abscess following an attack of typhoid fever some years before.

DR. ASHLEY speaks of the relation of lacerated cervix to salpingitis, etc. I cannot consider it a factor in the production of the disease, and regard it merely as a coincidence. If it were a potent factor in producing the trouble we should find salpingitis and pelvic adhesions far more frequently than we do now; for we must remember that in most women there is more or less laceration of the cervix during labor. Moreover this cause is certainly inapplicable to the frequent cases occurring in nulliparous women, and especially in virgins.

A close study of the clinical history of a number of cases inclines me to believe that the majority of cases follow infection during labor or after an incomplete abortion, for in many cases it is impossible to obtain even a history of leucorrhœa before the labor, which would apparently exclude gonorrhœal infection.

By infection during childbirth, I do not necessarily mean the cases in which we have well marked puerperal fever, but the milder degrees of infection as well; for most of the cases of so-called milk fever are due to infection and may give rise to serious results.

Zweifel, on the contrary, who has just published a remarkable series, 79 salpingo-oöphorectomies, with only one death, believes in the gonorrhœal origin of most cases. Sænger traces most of the cases in virgins back to a gonorrhœal salpingitis during childhood, which has persisted and ultimately affected the fallopian tubes. While I do not feel justified in subscribing to this view, I can say that it is quite probable.

For lately I have seen a number of cases of undoubted gonorrhœa in little girls of from two to seven years of age, in which there was no suspicion of criminal action.

In eight cases of vaginitis in little girls which I have examined, I found gonococci in six of them. In several, the mode of infection was quite clear. In one case the husband acknowledged an attack of gonorrhœa with which he infected his wife during her pregnancy, and each of the children born after it had ophthalmia neonatorum, followed, when they were older, by gonorrhœal vaginitis. In another case, an older brother had gonorrhœa and his two little sisters used his towels for bathing.

These remarks will show that the vaginitis of little children is not of strumous origin as is generally supposed, and that it demands a more active treatment than is generally employed, especially when we consider its possible consequences.

DR. BRINTON: I can corroborate the views of Dr. Williams, in regard to the specific origin of the cases of vaginitis in children, having recently treated first, the father with gonorrhœa, later the mother, and within a fortnight from the time the father consulted me was called to see the little daughter, aged four, with a severe "vaginitis" which yielded to the usual treatment in about the usual time. My experience has been that, if a child is found with a "vaginitis" close investigation will prove that some older member of the family has either "urethral" or "vaginal" discharge.

DR. NOBLE: Dr. Ashby has brought up so many points that it is difficult to know just what to take up.

It is now the fashion to call all unilateral collections of blood extra-uterine pregnancies. But I have recently had a case that proved not to be a pregnancy. With reference to the uterine hemorrhage coming from the tuber, we do know as a fact that it is possible for blood to come from the tuber. This was common to all in the days when the stump was treated by the extra-peritoneal method, in doing ovariotomy. I am quite sure that gonorrhœa has been the cause of most of the cases of pyosalpinx that I have seen. And I think that the cause of salpingitis in young women is often some simple infection. Many cases of dysmenorrhœa in young women are due to salpingitis. In such cases it is unnecessary to question their chastity. I agree with all the speakers in reference to the relation of lacerated cervix to salpingitis.

Where there is a laceration there is frequently an endometritis, and there is no reason to think that it may not follow out into the tube. I believe firmly in the great value of the drainage tube, and use it in almost every case. When properly cared for it is practically free from objection, while being of most positive advantage in allowing the escape of serum and blood.

DR. H. P. C. WILSON: I did an exploratory laparotomy for a fibro-cystic tumor. In manipulation I found great tendency to bleeding, and as I could not get at the ovaries nor remove the tumor without causing death, I closed the abdomen. She got on well for fourteen hours, when she became very feeble, heart and respiration very weak. She was put upon digitalis and muriate of quinine and urea, but it did no good. The heart became so weak that the pulse could not be felt. Then began with five minims of tincture of strophanthus every three hours, and ether m. xx, hypodermically every three hours. The pulse became stronger, 125 to the minute, and she felt better. The next day she became unconscious, pupils dilated, face flushed, pulse 120, temperature normal. The medicine was withdrawn, but she remained in this condition about twenty-four hours. To-day she is better, consciousness returning, pupils contracting. I have had no experience with the poisonous effects of strophanthus.

WILLIAM S. GARDNER, M. D., Secretary.

712 *N. Howard St.*

